# Diagnostic Accuracy of the Bethesda System for Reporting Thyroid Cytopathology (TBSRTC): An Institution Experience

**DOI:** 10.1155/2023/9615294

**Published:** 2023-11-15

**Authors:** Karima Rai, Joseph Park, Shamika Gokhale, Fatima Irshaidat, Gurdeep Singh

**Affiliations:** ^1^SUNY Upstate Medical University, 750 East Adams Street, Syracuse 13214, NY, USA; ^2^Lourdes Hospital, 161 Riverside Drive, Binghamton 13905, NY, USA

## Abstract

The Bethesda System for Reporting Thyroid Cytopathology (TBSRTC) is a standardized system which is used to classify results of thyroid fine-needle aspiration (FNA). This system is used to evaluate and determine which patients should get thyroid surgery. It was created in order to reduce the number of patients requiring surgery. The question remains as to whether this reporting system is accurate in determining those nodules that have malignant potential and those that do not. This study is a retrospective analysis of patients in one institution who have undergone FNA and then thyroid surgery based on TBSRTC. The outcome of the pathology reports after surgery was analyzed to determine the accuracy of TBSRTC in our institution (Lourdes Hospital, Binghamton, NY). The results from our institution were compared with similar studies in other institutions to determine accuracy and reproducibility. Our results indicated that the risk of malignancy in each Bethesda category was similar to the risk percentages described for most categories in the 2017 TBSRTC update.

## 1. Introduction

It is common to find thyroid nodules in the general population. Risk factors for nodule development include increased age, female gender (up to 4 times greater in females than males), and radiation exposure to the thyroid area [1, 2]. In addition, people who live in landlocked regions with a lack of iodine-fortified foods are at increased risk of thyroid nodule development [2]. Most thyroid nodules are benign, including thyroid adenomas (most commonly follicular adenomas), cysts, and goiters [2]. Thyroid cancer occurs in approximately 7 to 15% of nodules including thyroid carcinoma, thyroid lymphoma, or, rarely, metastasis (breast and kidney source) [3, 4]. Risk factors raising suspicion of thyroid cancer include radiation exposure in childhood (i.e., radiation therapy of the head and neck) and a family history of medullary/papillary thyroid carcinoma or familial tumor syndromes (i.e., Gardner syndrome and MEN2) [5].

According to the National Cancer Institute Surveillance, Epidemiology, and End Results (SEER) program, approximately 1.3 percent of people will be diagnosed with thyroid cancer during their lifetime and the rate of new cases of thyroid cancer is estimated at 14.6 per 100,000 people per year based on 2014–2018 data in the United States [6]. In 2019 alone, there were an estimated 915,664 individuals living with thyroid cancer in the United States [6]. The 2020 global incidence rate in women was estimated to be 10.1 per 100,000, which is approximately three times higher than that in men [7]. In addition, New York State's incidence rate of thyroid cancer was 19.6 per 100,000, which surpassed the national incidence rate of 14.1 per 100,000 [6]. This made New York the state with the highest incidence rate of thyroid cancer [6].

Evaluation of a palpable nodule begins with a thorough history and physical measurement of thyroid stimulating hormone (TSH) level, and performing an ultrasound of the thyroid [5]. Thyroid scintigraphy is then performed in patients with low or suppressed TSH, classifying the nodule as either cold or hot [5]. Fine-needle aspiration (FNA) of the nodule is indicated if a cold nodule is found on thyroid scintigraphy and signs of malignancy are present on ultrasound (irregular margins, taller than wide, marked hypoechogenicity, microcalcifications, rim calcifications with extracapsular growth, or extrathyroidal involvement) [5]. The size of the thyroid nodule is also considered when deciding on FNA of the nodule such that thyroid nodules less than 10 mm in size are monitored; however, the larger size of a thyroid nodule is not correlated with increased risk of thyroid cancer [3, 5]. Findings of FNA cytology (FNAC) are then categorized using the Bethesda system [8]. Standard management of thyroid cancer includes thyroidectomy and TSH suppression therapy [9]. Margins for excision are dependent on the stage/grade of the tumor [9]. Radioactive iodine ablation therapy (RAIA) is indicated for patients at high risk of cancer recurrence [9].

First formalized in 2007, the Bethesda System for Reporting Thyroid Cytopathology (TBSRTC) created a standardized nomenclature for thyroid FNA and provided malignancy risk for each of its six diagnostic categories [8]. Subsequently, there have been a number of enhancements to improve the TBSRTC, however, the original six categories remain unchanged (Table 1) [8]. Each diagnostic category has an implied risk of malignancy and recommended management guidelines (Table 1) [8]. TBSRTC has since been adopted in the United States with endorsement by the American Thyroid Association [5]. In addition, the TBSRTC provides two values for predicted risk in each category. One value includes noninvasive follicular thyroid neoplasm with papillary-like nuclear features (NIFTP) as being considered malignant while the other value considers NIFTP as nonmalignant (Table 1) [8].

Category III–V nodules are considered to be indeterminate or suspicious with 20–30% of nodules falling within these categories. Limitations exist as these nodules carry a wide range of malignancy rates, ranging from 6 to 75% on final pathology [10]. For example, patients with category III nodules with highly suspicious characteristics for malignancy are typically referred for surgery [4]. However, category III nodules that are found to have benign characteristics on repeat FNA must still be cautiously evaluated due to an underlying risk of malignancy [5]. Therefore, molecular markers have been employed to further risk-stratify nodules for surgical excision, specifically in categories III and IV. Commonly used tests include ThyGeNEXT/ThyraMIR v2, Afirma GSC (Afirma Gene Sequencing Classifier), and ThyroSeq v3 [11, 12]. These tests utilize genetic sequencing assays and molecular miRNA testing of the sample [13, 14]. Common mutations that are observed include those in the BRAF, V600E, and RAS genes [13, 14].

The main purpose of TBSRTC is to provide standardization in the nomenclature of thyroid nodules and recommended surgical and clinical management for such. There has been a growing concern that there is variability with the reporting of TBSRTC at different institutions. The purpose of this study is aimed at assessing the diagnostic utility of the 2017 TBSRTC at our institution with consideration of newer adjuvant diagnostic methods.

## 2. Materials and Methodology

### 2.1. Participants

The participants for this study were endocrinology patients seen by a single endocrinologist at Our Lady of Lourdes Memorial Hospital in Binghamton, NY. Patients included in this study were at least 18 years of age with thyroid FNA and pathology reports from this institution readily available. Only patients from this institution were assessed; however, some of the surgeries occurred at outside institutions. No pediatric patients were considered for this study.

### 2.2. Sample Size

581 patients were included in this study based on data availability. From these 581 patients, 855 thyroid nodules were aspirated, with multiple aspirations from certain patients depending on the size and character of the nodule as determined by the endocrinologist. Some patients also had repeat aspirations at later dates as their nodules grew and progressed.

### 2.3. Study Design and Protocol

This was a retrospective study with data collected from November 2015 through July 2020. Approval by Our Lady of Lourdes Memorial Hospital IRB was granted for this study. Data were collected by examining medical records of patients who had received a thyroid FNA at Our Lady of Lourdes Memorial Hospital. A Bethesda classification was reported by the pathologist in the pathology reports of the FNA encounters. In cases where no Bethesda classification was reported, the patient chart was reviewed by an endocrinologist, and the classification was manually entered into the data. Molecular marker testing with ThyraMIR was offered to all patients with nodules classified as Bethesda category I, III, and IV. In cases where surgery was performed, the tumor was assessed for malignancy and this assessment was compared with the initial Bethesda classification. This included surgeries performed outside of this institution. Only patients who received surgery on their nodules could have their diagnosis and Bethesda classification confirmed.

### 2.4. Statistical Analysis

Since malignancy could only be determined on nodules that were surgically excised, this leaves out potential malignancy in nodules not operated on. This explains the need to present a range of values for predicted malignancy. Based on the 2017 TBSRTC, the risk of malignancy (ROM) was presented as a range where the lower limit in each respective category is the number of malignant nodules/total number of nodules and the upper limit in each respective category is the number of malignant nodules/number of surgically excised nodules, where “malignant nodules” refers to confirmed malignancy upon surgical excision. Due to this range, it is believed that the true ROM lies between the two risk values [8, 15]. In addition, nodules found to be “noninvasive follicular thyroid neoplasm with papillary-like nuclear features” (NIFTP) were considered benign in this study. Since the introduction of NIFTP terminology in 2017, data before 2017 were reviewed to reclassify relevant cases as NIFTP.

## 3. Results

855 nodules were examined from 581 patients. 472 patients were female (81.24%), and 111 patients were male (19.1%). The average age of the patients was 55.8. 4.3% of all nodules were category I, 84.4% were category II, 5.2% were category III, 1.5% were category IV, 2.1% were category V, and 2.3% were category VI (Table 2).

Of the 855 nodules, 37 were found to be category I, 722 were category II, 44 were category III, 13 were category IV, 18 were category V, and 20 were category VI (Table 3). As listed in Table 3, surgery was performed on 1 nodule from category I, 24 nodules from category II, 5 nodules from category III, 10 nodules from category IV, 18 nodules from category V, and 19 nodules from category VI. The one category I nodule operated on was found to be malignant, and none of the category II nodules operated on were found to be malignant. Two of the category III nodules that were operated on were found to be malignant, two of the category IV nodules were malignant, 11 category V nodules were malignant, and 19 category VI nodules were malignant (Table 3). Table 3 lists the upper and lower limits for the overall ROM by Bethesda category at our institution, while Table 4 compares those values with the ROMs from other studies.

The percentage of patients choosing to undergo surgical resection also varied between the Bethesda classifications. Resection rates (RRs) varied from 2.7% in category I to 100% in category V (Table 5). The RR remained low in the lower-risk categories of II and III at 3.3% and 11.4%, respectively. The RRs increased at higher-risk categories of IV, V, and VI at 76.9%, 100%, and 95%, respectively (Table 5). The overall RR rate at our institution was 9%, which is lower than that of the other studies comparatively. Higuchi et al. had an RR of 56.3% but did not list individual RRs by Bethesda category (Table 5) [16]. Inabnet et al. started their study with candidates undergoing surgery, so their RR was 100% [17]. Nayar and Ivanovic had an overall RR of 27.2% with individual Bethesda category of RRs ranging from 10.7% to 88.2% (Table 5) [18]. Voung et al. did not give an overall RR but their individual Bethesda category of RRs ranged from 13% to 72.1% (Table 5) [19].

ThyraMIR is a molecular test for assessing oncogenic mRNA expression, which was used at our institution for certain patients with thyroid nodules classified as Bethesda categories I, III, and IV. There were 30 patients in total who underwent ThyraMIR testing. 28 patients tested negative for ThyraMIR (8 in category I, 18 in category III, and 2 in category 4), and 2 others had insufficient material and therefore testing was inconclusive. In addition, 2 patients in category I with negative ThyraMIR testing had negative ThyGeNEXT testing. All 30 patients who underwent molecular testing were able to avoid thyroidectomy/other surgical interventions.

## 4. Discussion

**Figure 1:**
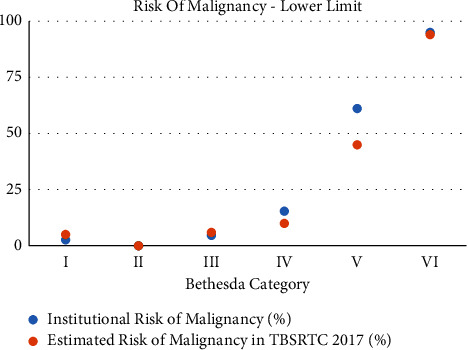
Lower limit of risk of malignancy in our institution and the 2017 TBSRTC.

**Figure 2:**
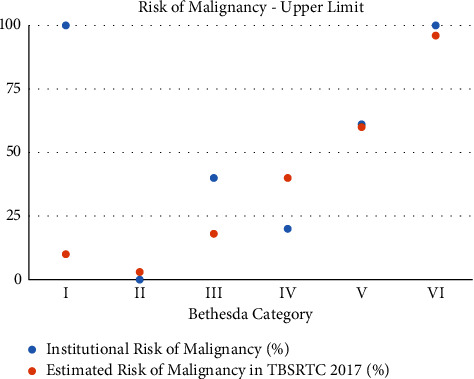
Upper limit of risk of malignancy in our institution and the 2017 TBSRTC.

Our results showed that, in our institution, the risk of malignancy in each Bethesda category was similar to the risk percentages described for most categories in the 2017 TBSRTC update. However, variability exists between the 2017 TBSRTC estimated ROMs and our study's ROMs in categories III, IV, and V (Figures 1 and 2). In addition, the upper limit of ROM in category I varies between the two studies and this is due to the nature of the category I classification being “nondiagnostic” due to insufficient sampling of the nodule [8]. The ambiguous nature of category I classification creates more variability, both in the number of patients choosing to undergo surgery, and the number of nodules ending up being malignant, hence the variability in the ROMs.

In the 2017 TBSRTC update, Cibas et al. mentioned the difficulty in capturing the true ROM in category III due to the small number of nodules that are excised [8]. In fact, the 2017 update recommends limiting the use of category III to less than or equal to 7% in all thyroid FNAs [8]. For our study, the percentage of total nodules placed in category III was 5.15%. The decision to excise the nodule is based on a variety of factors including molecular testing, patient preference, and clinical judgment. In order to account for the variability in category III of ROM, Cibas et al. suggest the use of an upper and lower limit [8]. As mentioned in the Methods section, the lower limit is the number of malignant nodules/total number of nodules and the upper limit is the number of malignant nodules/number of surgically excised nodules in each respective category [8]. The true ROM would lie in between the lower limit, which underestimates the ROM, and the upper limit, which overestimates the ROM [9]. In addition, Cibas et al. suggest that the true ROM for category III lies closer to 30% when NIFTP is considered as thyroid carcinoma; however, our study considered NIFTP as benign, not malignant [8].

With these considerations in mind, our institution's ROMs for categories III and IV still vary from the estimated ROMs described by Cibas et al. (Figures 1 and 2). In fact, they fall more in line with ROMs found in other studies for categories III and V, which are slightly higher than the TBSRTC 2017-estimated ROMs (Table 4). Studies used in comparison with our institution in Tables 2 and 4 include the work of Nayar and Ivanovic. Using data from a single institution in Chicago, they found that a six-tier reporting system for thyroid pathology was useful in guiding management, particularly in deciding whether surgery was needed [18]. Inabet, et al. utilized the registry data from across North America and Europe and concluded that ROMs in categories 1 to 4 were higher than expected than previously estimated ROMs in the first 2009 TBSRTC [17]. Voung et al. analyzed published thyroid pathology data from Western (America and Europe) and Asian countries. They found that ROM in Asian countries was higher than in Western countries throughout most diagnostic categories. In addition, they found that Asian countries had a smaller resection rate of their thyroid nodules than in Western countries [19].

The variability in ROM between our study and TBSRTC 2017 is seen best when comparing the upper limit of ROMs of categories III and IV (Figure 2). Since the upper limit of ROM is calculated by using the number of surgically excised nodules, the variability may be explained by the differences in management and indications for surgical excision between our institution and others. In addition, if more patients choose to have surgery on their nodules, the upper limit of ROM would be lowered if those nodules were confirmed to be nonmalignant. For example, in the study by Inabet et al. which looked at only surgically excised nodules, category III of ROM is less than our institution's category III of upper limit ROM [17]. In our study, only five out of the 44 category III nodules were operated on (11.4%), resulting in a wide ROM (4.6–40) which may explain the variability from TBSRTC 2017 data (Table 3). Although there is a variation, our institution now has a better understanding of the local malignancy rates, especially in category III nodules. This can guide our decision-making in whether to monitor nodules or refer for surgical excision.

**Figure 3:**
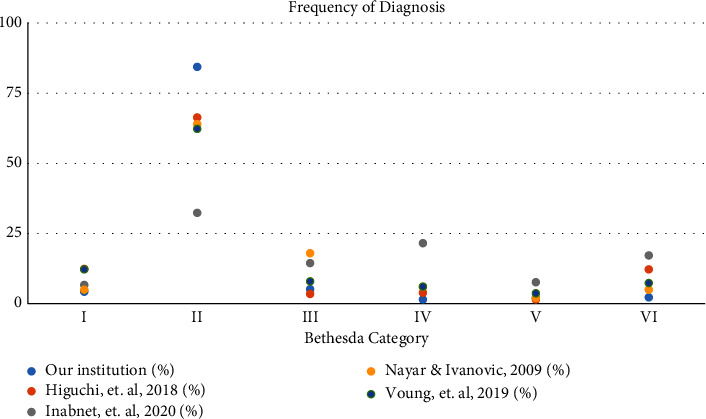
Frequency of diagnosis among different institutions.

The frequency of diagnosis in each Bethesda category for our institution also varies from other institutions and studies (Figure 3). Similar to several other studies of similar or larger sample sizes, a majority of nodules were found to be of category II; however, our study had the largest percentage of category II nodules and the lowest percentage of category VI nodules among the studies we looked at (Table 2). Variation can be attributed to the different rates of thyroid cancer that exist in these countries. Clinical decision-making and guidelines for the management of thyroid nodules may therefore differ between parts of the world in order to accommodate for the higher risk of thyroid cancer in that region. For example, the incidence of thyroid cancer is comparatively higher in East-Asian countries such as Japan and South Korea than in the United States [7].

**Figure 4:**
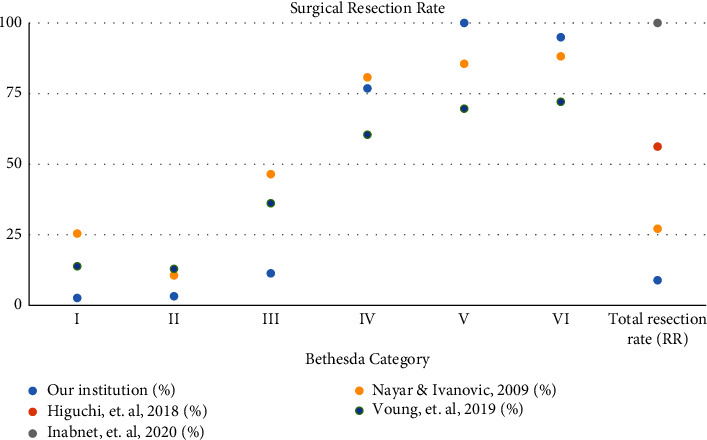
Surgical resection rates among different institutions.

In addition, when comparing the RRs between our institution and the other studies, our overall RR is much lower at 9%, with the next highest RR being 27.2% (Table 5). As shown in Figure 4, the lowest risk Bethesda categories I, II, and II had the lowest RRs both in our institution and comparatively in other institutions. As the risk increased (categories IV, V, and VI), our institution had higher RRs than others. This may have played a role in our variation from expected ROMs for each Bethesda category (Figure 3). This is due to the fact that malignancy can only be confirmed through surgical methods; therefore, studies with higher rates of surgery will likely have more accurate ROMs. Inabnet et al. started their study with patients undergoing surgery, therefore their RR was 100% [17]. While this skews the comparison of RRs between studies, it is helpful when comparing the calculated ROMs from this study with the others (Table 4). This is also why Inabnet et al.'s study did not have a lower limit of ROM; since everyone had surgery, all of those ROMs were of upper limit. However, even with an RR of 100%, the calculated ROMs from Inabnet et al.'s study still varied from the predicted ROMs in the TBSRTC guidelines (Table 4). For all but one Bethesda category, Inabnet et al.'s study had an estimated ROM higher than that of the TBSRTC upper limit of ROM. Since the TBSRTC did not report their RR, it is possible that this difference in ROMs between these two studies is also due to variability in the RR and the confirmation of diagnoses through surgery, or lack thereof.

As stated before, the decision to undergo surgery comes down to a variety of factors, such as expected risk, clinical guidelines, and shared decision-making between patient and provider. Our institution's low RR is due in part to the fact that the majority of our patients (84.4%) were classified in Bethesda category II, or benign, and likely decided that there was no need to undergo surgery, making the RR 3.3%. This varies from other institutions with category II RRs of 10.7% and 13% (Table 5). In addition, our category III RR was only 11.4%, while in other studies, it was 46.5% and 36.2% (Table 5). Variations in these RRs may be due to differences in clinical decision-making and guidelines in different parts of the world, as mentioned before [7].

The use of the Bethesda system in our institution has helped to accurately determine malignancy and guide further management. As seen in Table 3, the majority of nodules in category IV or beyond were surgically resected due to the higher risk of malignancy. Furthermore, all surgically removed nodules considered as category VI (malignant) were confirmed to be malignant. However, the results of our study vary when comparing the ROMs in categories III, IV, and V from the 2017 TBSRTC update and from our study. Currently, there is a lack of single institutional studies within the United States that assess the diagnostic utility and validity of the Bethesda system, particularly those following the 2017 TBSRTC update. While there are multiple studies outside of the United States investigating this particular question, the differences in thyroid cancer epidemiology make comparisons and evaluation of our study difficult. More studies within similar contexts are needed in order to see if this variability between institutional and estimated TBSRTC ROMs continues to exist. Then, we will be able to validate the diagnostic utility of the Bethesda categories within this region and more confidently use the Bethesda system to guide the management of thyroid nodules.

Considering the wide range of malignancy rates seen in category III–V nodules (6 to 75%), molecular testing is a useful and reliable tool to improve diagnostic certainty and allow for fewer surgical interventions [10]. The most commonly used molecular markers include ThyGeNEXT/ThyraMIR, Afirma GSC, and ThyroSeq v3. ThyGeNEXT/ThyraMIR is a molecular test assessing common genetic mutations seen in thyroid cancer and oncogenic mRNA expression, which was used at our institution for certain patients classified in the Bethesda categories of I, III, and IV. There were 30 patients in total who underwent ThyraMIR testing. 28 patients tested negative for ThyraMIR (8 in category I, 18 in category III, and 2 in category IV), and 2 others had insufficient material and therefore testing was inconclusive. In addition, 2 patients in category I with negative ThyraMIR testing had negative ThyGeNEXT testing. All 30 patients who underwent molecular testing were able to avoid thyroidectomy/other surgical interventions. The small sample size of 28 patients and the overall low risk of malignancy among Bethesda categories I, III, and IV (5 to 40%) may explain why all nodules submitted for ThyraMIR testing resulted as negative. As ThyGeNEXT/ThyraMIR has a negative predictive value (NPV) of 97%, it is a fairly reliable tool to improve the diagnosis of thyroid cancer [20]. In addition, based on hypothetical modeling, using molecular marker testing (ThyroSeq and Afirma GSC) was found to be more cost-effective in reaching the correct diagnosis than diagnostic lobectomy (surgery). The cost per correct diagnosis was $14,277 for ThyroSeq, $17,873 for Afirma GSC, and $38,408 for diagnostic lobectomy [21].

During the submission of this manuscript, the 2023 TBSRTC guidelines were released online in June 2023. Changes include the simplification of category titles and new ROMs for each category (Table 6); however, there still remain 6 categories of classification from nondiagnostic (category I) to malignant (category VI) [22]. Compared to the 2017 ROMs, the 2023 ROMs have increased for all categories [22]. The focus of this study is the comparison of our institutional data with the 2017 TBSRTC data. Future studies will need to be performed in order to further compare and analyze the 2023 data with our institution's data.

Multiple limitations exist for our study that may limit the generalization of our results. Our study was a retrospective chart review that lacked control for any variables. While all patients were of a single endocrinologist at one institution, the management of their FNA samples was not uniform. This includes multiple pathologists reading FNAs, and multiple surgeons performing surgery and surgical management. In addition, the decision-making to further surgically manage or observe nodules was not based on a standardized, single protocol. However, decision-making was mostly conducted by a single endocrinologist who also performed all the FNAs. This may help to decrease the effect of our study's limitation. Furthermore, we found that molecular testing was a helpful adjuvant in determining whether further management (i.e., surgery) was necessary. Therefore, further investigation is appropriate for molecular testing and its role in guiding the management of category III–V nodules.

## 5. Conclusion

TBSRTC seems to be fairly accurate when it comes to nodules that fall under category II or VI. Based on the data from our institution and the other comparative data, there appears to be more variability in ROM for Bethesda categories III–V, partially due to the nature of how these ROMs are calculated and the limitation of confirming diagnosis in patients without surgery. This variation in ROMs could still likely be the case when compared to the new 2023 classification as well. Additional studies are needed with similar RRs in order to compare ROMs between studies that have similar rates of confirmation of diagnoses through surgery. In addition, while there are multiple studies globally investigating this particular question, there is a lack of single institutional studies within the United States that assess the diagnostic utility and validity of the Bethesda System, particularly those following the 2017 TBSRTC guidelines, and now the 2023 TBSRTC update. Therefore, more studies within similar contexts are needed to truly validate the diagnostic utility of the Bethesda categories within this region and the diagnostic utility of the 2023 TBSRTC guidelines as well.

## Figures and Tables

**Table 1:** TBSRTC 2017 Bethesda categories [8].

Bethesda category	Category title	Risk of malignancy if NIFTP = cancer (%)	Risk of malignancy if NIFTP ≠ cancer (%)
I	Nondiagnostic or unsatisfactory	5–10	5–10
II	Benign	0–3	0–3
III	Atypia of undetermined significance (AUS) or follicular lesion of undetermined significance (FLUS)	6–18	10–30
IV	Follicular neoplasm or suspicious for a follicular neoplasm	10–40	25–40
V	Suspicious for malignancy	45–60	50–75
VI	Malignant	94–96	97–99

**Table 2:** Frequency of diagnosis for each Bethesda category.

Bethesda category	Our institution (%) *N* = 855	Higuchi et al. (%) [16] *N* = 10,399	Inabnet et al. (%) [17] *N* = 21,746	Nayar and Ivanovic (%) [18] *N* = 5194	Voung et al. (%) [19] *N* = 145,066
I	4.3	12.4	6.7	5	12.2
II	84.4	66.4	32.4	64	62.3
III	5.2	3.5	14.5	18	8.0
IV	1.5	3.9	21.6	6	6.1
V	2.1	1.5	7.7	2	3.7
VI	2.3	12.2	17.2	5	7.4

**Table 3:** Result of surgeries on thyroid nodules.

Bethesda category	Number of nodules	Number of nodules operated on	% of nodules operated on, resection rate (RR)	Number of nodules found to be malignant	Risk of malignancy overall (%), lower limit	Risk of malignancy of nodules operated on (%), upper limit
I	37	1	2.7	1	2.7	100
II	722	24	3.3	0	0.0	100
III	44	5	11.4	2	4.6	40
IV	13	10	76.9	2	15.4	20
V	18	18	100	11	61.1	61.1
VI	20	19	95	19	95.0	100

**Table 4:** Risk of malignancy for each Bethesda category.

Bethesda category	Institutional risk of malignancy (%) *N* = 855	Estimated risk of malignancy in TBSRTC (%) [8]	Risk of malignancy in Inabnet et al. (%) [17] *N* = 21,746	Risk of malignancy in Nayar and Ivanovic (%) [18] *N* = 5194	Risk of malignancy in Voung et al. (%) [19] *N* = 145,066
I	2.7–100	5–10	19.2	9	2.0–19.1
II	0	0–3	12.7	2	0.7–8.0
III	4.6–40	6–18	31.9	6	9.2–30.5
IV	15.4–20	10–40	31.4	14	28.9
V	61.1	45–60	77.8	53	79.6
VI	95–100	94–96	96.0	97	99.1

**Table 5:** Rates of surgical resection between institutions.

Bethesda category	Our institution (%) *N* = 855	Higuchi et al. (%) [16] *N* = 10,399	Inabnet et al. (%) [17] *N* = 21,746	Nayar and Ivanovic (%) [18] *N* = 5194	Voung et al. (%) [19] *N* = 145,066
I	2.7	N/A	N/A	25.5	13.9
II	3.3	N/A	N/A	10.7	13
III	11.4	N/A	N/A	46.5	36.2
IV	76.9	N/A	N/A	80.8	60.5
V	100	N/A	N/A	85.6	69.7
VI	95	N/A	N/A	88.2	72.1
Total resection rate (RR)	9.0	56.3	100	27.2	N/A

**Table 6:** TBSRTC 2023 Bethesda categories [22].

Bethesda category	Category title	Risk of malignancy if NIFTP = cancer (%)	Risk of malignancy if NIFTP ≠ cancer (%)
I	Nondiagnostic	5–20	12
II	Benign	2–7	2
III	Atypia of undetermined significance (AUS)	13–30	16
IV	Follicular neoplasm	23–34	23
V	Suspicious for malignancy	67–83	65
VI	Malignant	97–100	94

## Data Availability

Data can be provided to any person/third party from the corresponding author upon request for up to 2 years after publication.
